# Why Do Employers (Fail to) Hire People with Disabilities? A Systematic Review of Capabilities, Opportunities and Motivations

**DOI:** 10.1007/s10926-022-10076-1

**Published:** 2023-01-23

**Authors:** Rosanna Nagtegaal, Noortje de Boer, Rik van Berkel, Belle Derks, Lars Tummers

**Affiliations:** Utrecht School of Governance, Utrecht University, Bijlhouwerstraat 6, 3511 ZC Utrecht, The Netherlands

**Keywords:** Hiring, Employers, People with disabilities, Systematic review, COM-B

## Abstract

**Purpose:**

To increase the number of people with disabilities in employment, we need to understand what influences employers’ hiring decisions. In this systematic review, we map out factors affecting employers’ hiring decisions about people with disabilities.

**Methods:**

This study is a systematic review that applies the COM-B model to identify factors that contribute to employers (not) hiring people with disabilities. The COM-B model proposes that employers will perform hiring behavior (B) if they have the capability (C), opportunity (O) and motivation (M) to do so. We also investigate if factors have a negative, positive or no effect. We report in accordance with the PRISMA guidelines.

**Results:**

In a review of 47 studies, we find 32 factors. Most of these factors are barriers. The most frequently mentioned barriers are employers’ (1) expectations that people with disabilities are unproductive, (2) expectations that people with disabilities cost a lot of money, and employers’ (3) lack of knowledge about disabilities. The most researched facilitators for employers to hire people with disabilities include (1) the motivation to help others, (2) working in a large organization, and (3) expecting a competitive advantage. The effect of factors can differ depending on contextual circumstances, including the type of organization, the type of disability and different policies.

**Conclusions:**

We conclude that hiring decisions are influenced by an array of different barriers and facilitators. The effect of these factors can differ across organizations and disability types. Our study of factors affecting hiring can be used by scholars, policy makers, and organizations to create interventions to increase the hiring of people with disabilities.

**Supplementary Information:**

The online version contains supplementary material available at 10.1007/s10926-022-10076-1.

Approximately 15 percent of the world’s population, hence more than 1 billion people, live with some form of disability [1]. At the same time, employment rates for people with disabilities are much lower than those for people without disabilities. The 2021 Joint Employment Report of the EU, for example, reports an employment gap of 24% across EU countries when comparing employment of people with and without disabilities [2]. This inequality in the distribution of employment is important to address because unemployment can lead to poverty [1]. On top of that, employment can improve social inclusion and human dignity. Many countries are taking measures to promote the labor-market participation of people with disabilities. Countries have, for instance, adopted measures to increase employment rates, such as anti-discrimination laws and quotas. Regardless, employment of people with disabilities remains low [3].

Social policies aimed at promoting the labor-market participation of people with disabilities have traditionally mainly focused on the supply side by targeting people with disabilities. Policies thus targeted the motivations, skills, and capabilities of people with disabilities to make them more attractive for employers [4]. An example is that well designed vocational rehabilitation programs for people with a disability can increase employment [5, 6]. More recently, however, policies that combat unemployment of people with disabilities have started to focus on the demand-side of the labor-market by targeting employers and their organizations. For example, the UN Convention on the Rights of Persons with Disabilities that became effective in 2008 stimulated the introduction of anti-discrimination legislation in many countries [7]. This demand-side orientation in social policies increases the relevance of knowledge of what drives employers to (not) hire people with disabilities and what barriers and facilitators play a role [8].

There are numerous theories aimed at understanding changes in behavior, such as hiring. These theories are however diverse, complex and can even overlap [9]. Therefore, Michie et al. [9] created a comprehensive model that bundles theories used to understand changes in behavior into the ‘COM-B model’. The COM-B model proposes that for people to perform a certain behavior (B) they need capability (C), opportunity (O) and motivation (M). This model has been widely applied to understand behavior and pinpoint interventions that can change behavior [10, 11]. We use the COM-B model to understand employers’ hiring behavior of people with a disability. Therefore, our research question is: *Which capabilities, opportunities and motivations of employers impact whether they hire people with disabilities?*

We answer this research question by conducting a systematic literature review. We contribute to existing literature in three ways. First, we focus specifically on hiring behaviors by employers. Scholars have so far mainly focused on intentions to hire as an outcome variable [12] rather than on the actual hiring behavior of employers. While insights into intentions to hire are valuable, these do not necessarily translate into hiring behavior. There is a notable intention-behavior gap. In an overview article, Sheeran and Webb [13] conclude that intentions translate into action one-half of the time. We therefore focus on understanding hiring behavior instead of intentions.

Second, we are—to the best of our knowledge—the first to use the COM-B model to understand hiring behavior of employers. While scholars have started to investigate which factors drive employers to hire people with disabilities, scholars primarily dissected employer attitudes (see Burke et al. [14] for a systematic review). Few studies have used theoretical models of behavior to analyze these factors. In this study we use the COM-B model. The COM-B model explains that *capabilities, opportunities* and *motivations* are factors that influence behavior. The COM-B model can be used to conduct a behavioral analysis [9] and has already been used to identify factors influencing behavior in various settings [11, 15–17]. It has, however, not yet been applied to hiring behaviors. Our behavioral analysis contributes to evidence-based policy measures directed at employers such as campaigns and quotas [3]. It can also offer avenues for novel interventions as every COM-B component is connected to specific interventions through the Behavior Change Wheel [9]. Our study can thus identify factors that could help policy makers create interventions that reduce barriers or amplify facilitators and, ultimately, stimulate employers to hire more people with disabilities.

Third, and finally, we add to literature by including a systematic overview of capabilities, opportunities and motivations with a positive, negative or no effect on hiring behavior. We also distinguishing these factors across different types of disabilities. Other reviews have focused on either the negative side [18] or the positive side [19] of hiring people with disabilities, or both [20, 21]. The field, however, lacks a systematic overview in which positive, negative and no effects on hiring behavior are mapped per factor. Here, the inclusion of null effects is especially important as they are often overlooked yet informative [21]. We also study whether factors work differently for disability types, being psychological, physical and intellectual disability. This can help policy makers to create effective interventions while taking important contextual factors into account.

## Theoretical Framework

### People with Disabilities

The International Classification of Functioning, Disability and Health (ICF) defines disability as an umbrella term for impairments, activity limitations and participation restrictions. It denotes the negative aspects of the interaction between an individual (with a health condition) and that individual’s contextual factors [22, p.8]. This means that disabilities are not only a result of bodily functions and structures (the medical model of disability), but also a result of a person being unable to operate within an environment (the social model of disability) [23]. For example, when a blind person (physical impairment) does not have to use sight for a job (activity and participation restrictions), this person is not considered disabled in that context. We use the ICF definition because it is the most used definition for disability [24, 25] and includes medical as well as social aspects of disability. This definition is in line with the UN Convention on the Rights of People with Disabilities [7, p. 1] that stipulates in its preamble that “disability results from the interaction between persons with impairments and attitudinal and environmental barriers that hinders their full and effective participation in society on an equal basis with others”. Finally, the ICF definition fits with the increasing demand-side orientation in social policies mentioned above that focus on attitudinal and environmental barriers in the workplace.

### The COM-B Model

**Fig. 1:**
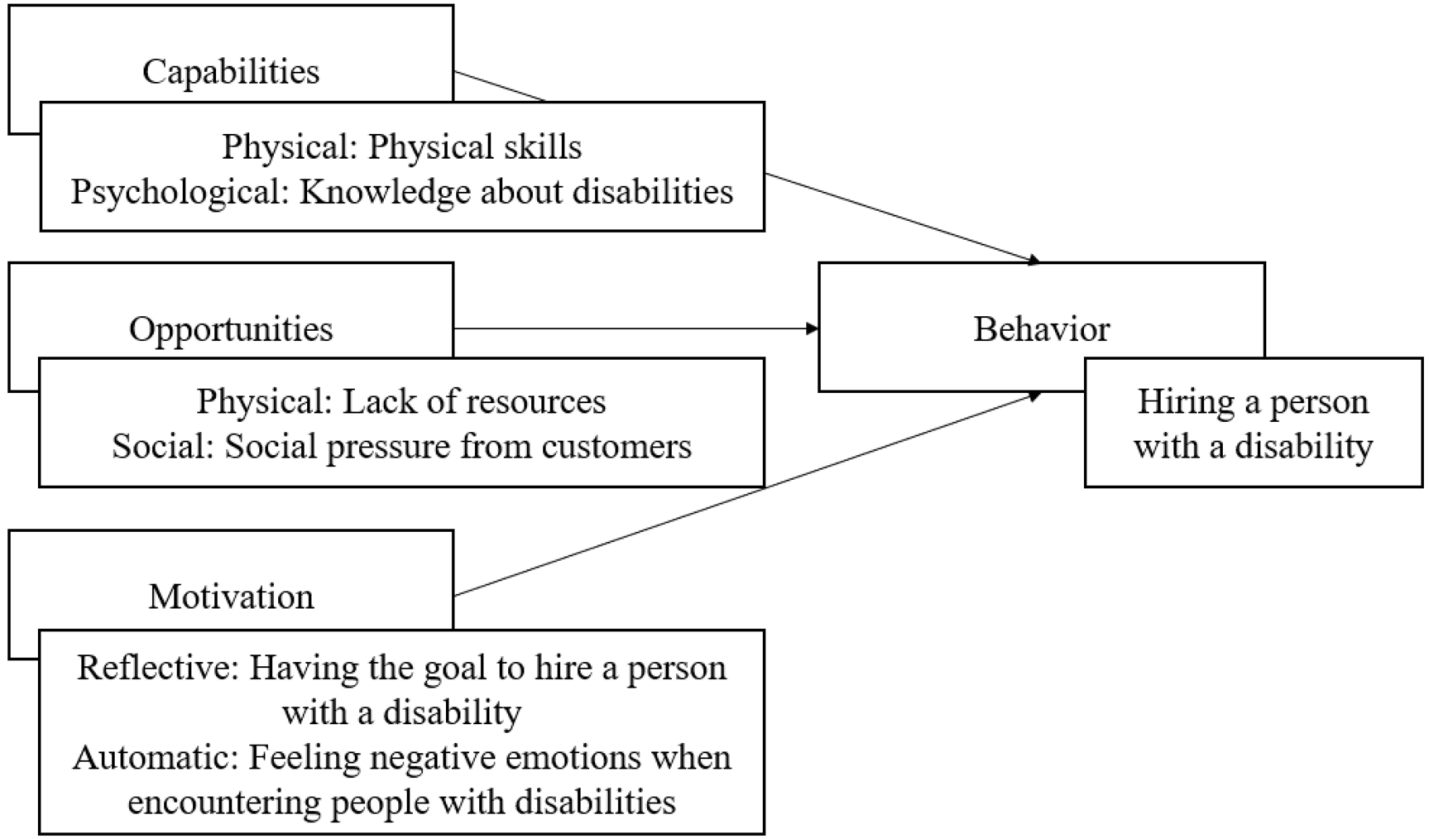
The COM-B model with dimensions and examples

The aim of this review is to identify capabilities, opportunities and motivations of employers that influence hiring people with disabilities. Many different theories about how psychological factors affect decisions exist, including the theory of planned behavior [26] and self-efficacy theory [27]. These theories have been bundled into different models [28]. One of these models is the COM-B model. The COM-B model explains that Capability, Opportunity and Motivation (COM) influence Behaviors (B). Capabilities can be physical (for instance physical skill, strength or stamina) or psychological (cognitive knowledge or skills, strength or stamina). Opportunity can be physical (influences from the environment including time, resources, locations) and social (interpersonal influences such as social cues, group pressures and cultural norms). Motivation can be reflective (processes involving plans and evaluations) and automatic (emotional reactions, desires, impulses etc.) [9, p. 63]. Figure 1 shows the COM-B model with examples.

The COM-B model has benefits over other models that aim to understand behavior [11, 28]. First, the COM-B model has a strong theoretical underpinning. Connected to the COM-B model is the Theoretical Domains Framework, which bundles 33 behavioral theories into 14 domains [29, 30]. An overview of the Theoretical Domains Framework is shown in the Supplementary Information. Second, the model is linked to the Behavior Change Wheel, which is specifically aimed at designing interventions to change behavior [31]. This is important for the research field of rehabilitation, as one clear goal is to increase the hiring of people with disabilities. Third, the model includes environmental and social factors, which in the context of hiring people with disabilities is relevant. These factors refer for instance to social pressures that an employer experiences, but also the regulations that an employer has to deal with.

### Hiring Behavior

This review focuses on which factors impact hiring people with disabilities. This means that we concentrate on actual hiring behaviors. We refer to hiring behavior and decisions as behavior and decisions made by *employers*, which is common practice in the literature. However, ‘employers’ behavior and decisions’ in practice are actually a set of diverging operations within the hiring process by many different actors such as HR departments, supervisors, managers and teams. The decisions and behaviors of these actors are often structured by official organizational personnel and HRM policies. Thus, in many cases hiring behavior is not just employer behavior, nor is it individual behavior. We, therefore, consider the whole variety of organizational actors as hirers.

Several studies have investigated factors related to the COM-B model that affect hiring. Kaye, Jans, and Jones [32] for instance show that lack of awareness of disability and accommodation issues, concern over costs, and fear of legal liability affect decisions to hire. Bonaccio et al. [18] show that employers often have negative stereotypes of people with disabilities. Employers for instance could believe that people with disabilities are less productive. Employers can also believe that providing accommodations is costly. We however do not have an overview of factors relating to the COM-B model.

## Methods

We report in accordance with the PRISMA guidelines [33]. Our strategy consists of four steps [34]. First, we search the databases PsycINFO, Web of Science, Scopus and PubMed for studies using search terms that cover ‘hiring’ and ‘disabled people’ and related terms. The full list of search terms is included in the Supplementary Information. The databases were selected based on earlier systematic reviews as well as a consultation with a university librarian. Scopus and Web of Science are general databases that include sociological and business journals. Furthermore, PsycINFO is the primary database for psychological studies. We selected a number of key articles to help identify the effectiveness of the search.

For this search, we selected our articles with the ASReview tool [35]. This is a novel machine learning tool aimed at selecting articles for full text review from database searches by sorting articles based on relevance. ASReview was developed by researchers of Utrecht University to help scientists provide timely and transparent systematic reviews. During its development, ASReview’s performance was assessed by conducting simulations [35]. In the current study, two coders reviewed the first 10% of identified articles. Afterwards we aimed to either exclude 100 articles sequentially or review 36%. The latter percentage was based on the most conservative estimation from a simulation study [36]. We reached the point of 100 sequential exclusions after reviewing 26%. This same technique was also applied to the second step of our strategy, which was a search specific journals. We searched in the three journals: *Journal of Occupational Rehabilitation, Journal of Vocational Rehabilitation* and *Rehabilitation Counseling Bulletin*. These journals provided the most articles through our database search and had a search function available. For the database search we limited ourselves to titles. For the journal search we expanded our search to all fields. For the journal search, we reached 100 sequential exclusions after reviewing 15.1%.

**Fig. 2:**
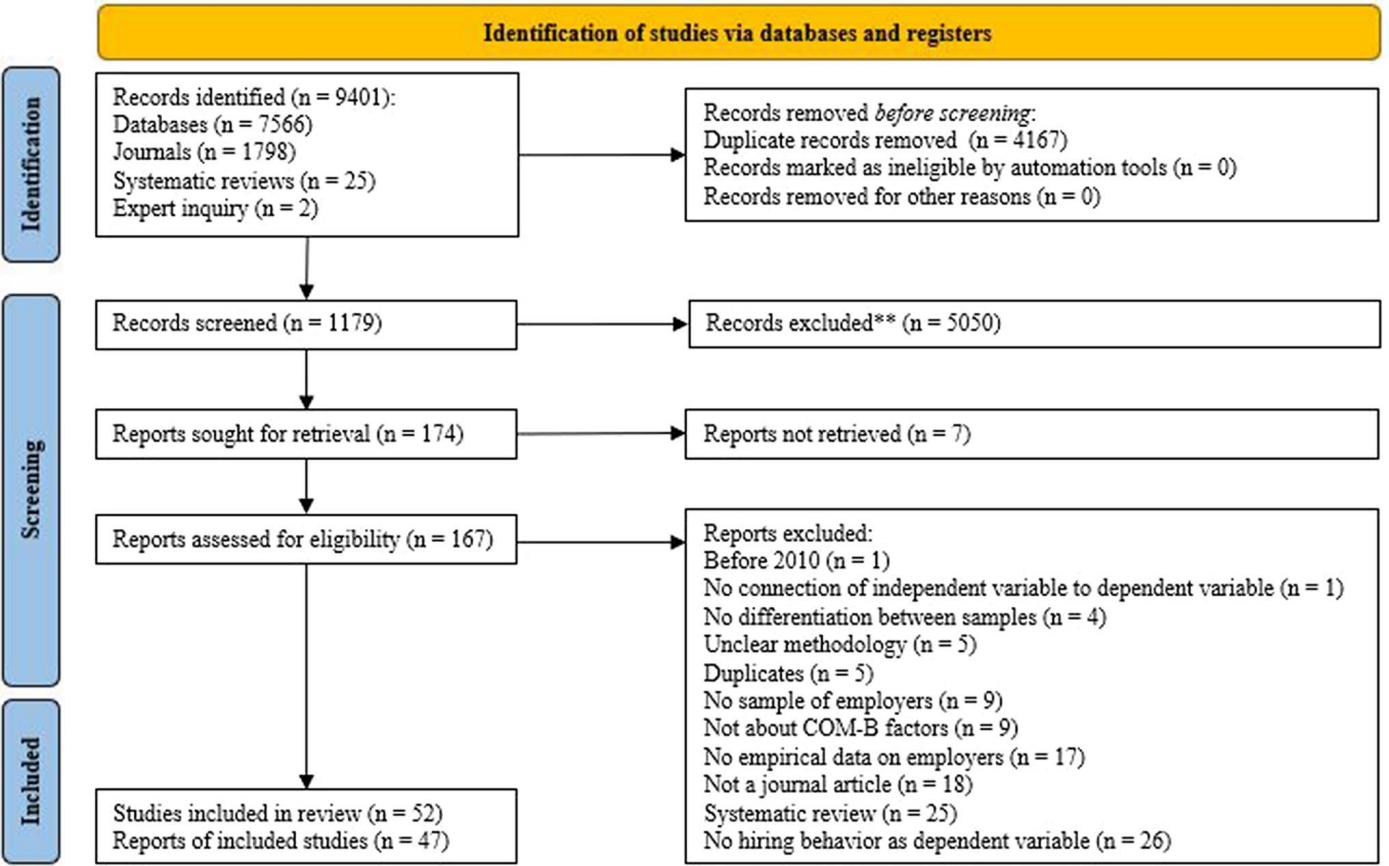
PRISMA flow chart. **ASReview were used to screen the data. We screened (n = 881, 25.95%) of the database search and (n = 271, 15.1%) of the journal search

Third, we scanned the reference list of 5 earlier systematic reviews on the topic of interest that we found either in the database or journal searches. Fourth, we asked 9 experts to add relevant studies to the studies we had selected. Experts suggested two studies, which were in the end excluded. The full process of identifying studies is visible in Fig. 2. We did not preregister our review. Yet, to ensure transparency, we provide open data, from the search results to the coding. Our data is available on Open Science Framework (see https://osf.io/av95r/).

We included studies if they met the following criteria:

- *Type of study and participants:* Studies had to be about employers and focus on factors related to the COM-B model influencing hiring behaviors regarding people with disabilities.
- *Study design:* The studies had to be empirical. We included qualitative as well as quantitative studies.
- *Publication status and language:* Studies had to be published in journals, peer-reviewed and written in English.
- *Year of publication:* Studies reported in articles published from 2010 onwards. The search ended on 22-02-2022.

We coded factors into the Theoretical Domains Framework (TDF). Factors were later placed in the COM-B framework as described by Michie et al. [9]. We used this stepwise approach as we wanted the coding of factors to be as detailed as possible. We, therefore, chose to start with the detailed TDF and then re-cluster our codes into the COM-B framework. For example, we first coded the factor ‘not knowing how to manage people with disabilities’ into the TDF category ‘knowledge’ which falls under the COM-B category ‘psychological capability’. We also coded different types of disabilities (physical, psychological and intellectual) and types of companies (public/private). For a measure in size of the organization we used the EU definition regarding small and medium-sized enterprises (SMEs). This demarcates micro companies (< 10 employees), small companies (< 50), medium-sized companies (< 250) and large companies (> 250) [37]. We also coded for the journals, the authors and countries the studies were conducted in. This was done by one coder. Difficult cases were discussed.

## Results

### Finding 1: Distribution of the Studies

We included 47 articles in our study. We found that most studies were conducted in Western countries such as the United States of America (40%), Australia (9%), the Netherlands (4%) and Sweden (4%). Some studies were conducted in non-Western countries such as South-Africa (6%), and China (4%). Studies were most often qualitative in nature (62%) and used interviews (47%). Other studies were quantitative (47%) and mostly used survey methodology (31%). Experiments were rarely used (6%). Articles were most often published in Journal of Vocational Rehabilitation (15%), Journal of Occupational Rehabilitation (9%) and Rehabilitation Counseling Bulletin (9%). We also found that most studies investigated companies of multiple sizes (43%), did not clarify the type of disability they addressed (64%), and included both public and private employers (58%).

### Finding 2: Hiring Behaviors Mostly Measured Subjectively

Table 1 shows an overview of the dependent variables used to measure hiring behaviors in the articles. Measures of hiring varied substantially. We found that most measures of hiring were subjective. This means that employers reported their thoughts on what influenced hiring behavior, without data on the actual amount of people with disabilities hired being reported. Of these subjective measures, some studies focused on hiring of people with disabilities by employers in general (7%). This means employers reflected about what factors influenced employers hiring in society. These studies for instance asked about what challenges employers can experience in hiring people with disabilities [32]. Although these studies do not measure actual behavior, the benefit of asking about hiring of other employers is that it can account for social desirability bias. Other studies focused specifically on hiring people with disabilities in their own organizations (47%). This was at times abstract, and for instance about hypothetical hiring (i.e. what *would* influence *your* hiring behavior). Some studies used more objective hiring measures. These studies for instance measured whether employers were actively recruiting people with disabilities (5%) or called people back for interviews (2%). Studies sometimes also referred to real accounts of hiring in the past (25%).

**Table 1:** Measurements of hiring behaviors

Measurement	Number (%)	Example [reference]
*Subjective*		
Hiring of people with disabilities in general by employers	4 (7%)	Asking for proposed reasons for employers (third-parties) not to hire people with disabilities [32]
(not) Hiring of people with disabilities in their company	28 (47%)	Asking for perceptions of barriers to the employment of people with disabilities within their organizations [37]
Reported actively recruiting	3 (5%)	Percent actively recruiting people with disabilities as reported in survey [38]
Reported hiring of employees with disabilities	4 (7%)	Reported percent of people with disabilities in the company’s workforce [39]
The perceived extent of hiring people with disabilities	1 (2%)	4-point Likert type scale on the extent to which people with disabilities are hired [40]
*Objective*		
Call back for interview	1 (2%)	Employers expressing desire for an interview [6]
Employer expressing interest in candidate	1 (2%)	Employers expressing other interest than an interview, for instance by asking for more credentials [6]
Hypothetical hiring in an experiment	2 (3%)	A scale from 1 to 10 about whether the fictitious candidate would be offered an interview [41]
Having hired in the past or having employed currently	15 (25%)	Reflecting on real past hiring accounts by including current employers of people with disabilities [42]
Total	59 (100%)	

*Note*. Studies can contain multiple dependent variables

### Finding 3: Beliefs About People with Disabilities not Being Productive and Expectations About Costs Mentioned Most in Literature

In total, we identified 32 factors that can influence hiring behavior. These factors were reported 421 times in total. Some factors were recoded for interpretation purposes. For example, in the factor ‘lacking knowledge about recruiting’ we also included recoded instances where ‘knowledge about recruiting’ was mentioned. Effects of these factors were recoded as well. In the Supplementary Information we have reported which percentage of factors is recoded.

In Tables 2, 3 and 4 we show the capabilities, opportunities and motivations that were mentioned in the literature. We indicate whether they were mentioned as having a negative, positive or no effect. Overall, we found that employers’ beliefs about people with disabilities not being productive (14%) and employers’ expectations about costs (11%) were mentioned the most in literature. After that, having pro-social motivation, not knowing how to manage people with disabilities, lacking knowledge about disabilities and experiencing administrative burden were mentioned the most (5%). Most factors fall into the motivation category of the COM-B model. In the Supplementary Information, we have defined all factors.

**Table 2:** Capabilities influencing hiring behaviors

	Negative	No effect	Positive	Total
*Capabilities (psychological)*				
Lacking knowledge about recruiting	5 (71%)	2 (29%)	0	7 (2%)
Believing negative stereotypes	6 (55%)	5 (45%)	0	11 (3%)
Not knowing how to manage people with disabilities	12 (60%)	8 (40%)	0	20 (5%)
Lacking knowledge about disabilities	16 (71%)	5 (24%)	0	21 (5%)
Total	39 (66%)	20 (34%)		59 (14%)

*Note*. The percentages accompanying the effect are in relationship to one factor. The percentages in the right column represent how many times the factor is mentioned/all mentions of factors

**Table 3:** Opportunities influencing hiring behaviors

	Negative	No influence	Positive	Total
*Opportunities (social)*				
Believing co-workers will respond negatively	9 (82%)	2 (18%)	0	11 (3%)
Believing customers will respond negatively	4 (100%)	0	0	4 (1%)
Not having support from within the organisation	5 (100%)	0	0	5 (1%)
*Opportunities (physical)*				
Working in a worksite with physical barriers	5 (71%)	2 (29%)	0	7 (2%)
Working for a public organisation	0	6 (75%)	2 (25%)	8 (2%)
Getting financial incentives	0	1 (8%)	11 (92%)	12 (3%)
Not encountering qualified people with disabilities applying	10 (77%)	3 (23%)	0	13 (3%)
Working in an organisation with a policy for inclusion	0	1 (8%)	12 (92%)	13 (3%)
Having positions available	0	2 (14%)	12 (86%)	14 (3%)
Lacking external support	14 (93%)	1 (7%)	0	15 (4%)
Working in a large organization	0	2 (12%)	14 (88%)	16 (4%)
Experiencing administrative burden	17 (89%)	2 (11%)	0	19 (5%)
Total	64 (47%)	22 (16%)	51 (37%)	137 (33%)

*Note*. The percentages accompanying the effect are in relationship to one factor. The percentages in the right column represent how many times the factor is mentioned/all mentions of factors

**Table 4:** Motivations influencing hiring behaviors

	Negative	No influence	Positive	Total
*Motivation (reflective)*				
Expecting limited consequences of hiring	0	0	1 (100%)	1 (0%)
Being willing to take a risk	0	0	4 (100%)	4 (1%)
Feeling in control	0	1 (33%)	2 (67%)	3 (1%)
Lacking intentions to hire	2 (67%)	1 (33%)	0	3 (1%)
Expecting financial gains	0	0	7 (100%)	7 (2%)
Expecting negative safety consequences	6 (86%)	1 (14%)	0	7 (2%)
Worrying about spending more time to assist	7 (100%)	0	0	7 (2%)
Believing people with disabilities have unique advantages	0	0	8 (100%)	8 (2%)
Worrying about legal consequences	10 (67%)	5 (33%)	0	15 (4%)
Expecting a competitive advantage	0	1 (6%)	15 (94%)	16 (4%)
Having pro-social motivation	0	2 (9%)	20 (91%)	22 (5%)
Expecting costs	26 (57%)	20 (43%)	0	46 (11%)
Believing people with disabilities are not productive	50 (82%)	11 (18%)	0	61 (14%)
*Motivation (automatic)*				
Experiencing positive emotions	0	0	1 (100%)	1 (0%)
Experiencing negative emotions	6 (55%)	4 (36%)	1 (9%)	11 (3%)
Complying with laws	0	4 (31%)	9 (69%)	13 (3%)
Total	107 (48%)	50 (22%)	68 (30%)	225 (53%)

*Note*. The percentages accompanying the effect are in relationship to one factor. The percentages in the right column represent how many times the factor is mentioned/all mentions of factors

In Table 2 above we see that for capabilities the focus is mostly on the lack of knowledge about different topics related to hiring people with disabilities. The factor mentioned most was that a lack of knowledge about disabilities can be a barrier (5%). An example is that employers are more reluctant to hire someone with an unknown disability type [43]. We found no mention of physical capabilities influencing employers.

For social opportunities, as shown in Table 3, the most mentioned factor was that employers believe co-workers will respond negatively to people with disabilities (3%). We also find that physical opportunities were mentioned in literature. The most common factor for the physical opportunities category was experiencing administrative burden (5%). This was predominantly a barrier (89%). Administrative burden refers to those burdens that citizens, or in this case employers, experience when interacting with the state [44]. We found that studies referred to, for instance, excess bureaucracy [45], limited help in finding applicants [46] and a lack of understanding of the rules [47]. After that, the most reported facilitator (in 89% of cases) was working in a large organization (4%). This can be because large organizations have more resources, such as a specialized diversity manager, that contribute to hiring people with disabilities [46].

Table 4 shows which motivations influence hiring behavior. The most researched motivational factor was that employers believe that people with disabilities are not productive (14%). An example is that employers expect that people with disabilities will have more work-related performance difficulties [48]. This was sometimes related to the nature of the work. To illustrate, Huang and Chen [43] showed that some employers mentioned they did not hire applicants in wheelchairs because the jobs required standing and moving. Beliefs about people with disabilities not being productive had a predominantly negative effect on hiring behavior (82% negative, 18% neutral, 0% positive).

The second most mentioned factor was that employers expect costs when hiring a person with a disability (11%). Sometimes, these concerns were abstract. For instance, Houtenville and Kalargyrou [49] showed that employers mentioned that it ‘costs more to employ people with disabilities’. More specifically, these cost concerns were related to, for instance, accommodation or supervision (see for example Ta et al. [50]). This factor had a negative (57%) as well as no effect (43%) on hiring. In the category motivation, we also found that pro-social motivation was often mentioned as a facilitator (5%). Pro-social motivation is defined as the desire to contribute to the well-being of others [51]. Concerning hiring people with Autism Spectrum Disorder, employers for instance noted that every person has a right to work [52]. This factor had positive effects (91%) on hiring behavior. Another factor that was mentioned often as a facilitator (94%) was expecting a competitive advantage (4%). The inclusion of people with disabilities can for instance help strengthen the brand of the organization [48].

We also identified a number of factors that were rarely discussed, these factors were ‘expecting limited consequences of hiring’ (1%) ‘lacking intentions to hire’ (1%) ‘feeling in control’ (1%), and ‘experiencing positive emotions’ (0%, rounded). We are unable to conclude if the lack of reporting on these factors is because they are insufficiently researched or just not found by researchers.

### Finding 4: A Factor Does not Always Have the Same Effect on Hiring Behavior

Tables 2, 3 and 4 show that not all factors have a clear-cut influence on hiring behavior. The direction of the effect on hiring (negative, positive or no effect) can vary for the same factor. To illustrate, factors often had positive or negative effects on hiring while they were also reported to sometimes have no effect. For example, this was found for the factor ‘having positions available’. Some employers mentioned that for people with intellectual disabilities technology has reduced the need for manual completion of certain tasks [53]. Other employers were reported to apply a strength-based, person-centred approach and were able to able to identify more employment opportunities [46]. In other words, the factor ‘having positions available’ has mixed effects on hiring people with disabilities.

On top of that, some of these factors seem to contradict each other. For instance, ‘expecting costs’ (11%) and ‘expecting financial gains’ (2%) seem polar opposites. This means that some employers are not concerned about costs associated with hiring people with disabilities but instead see financial gains from hiring, for instance because of low turnover [43]. This was found also for ‘believing people with disabilities are not productive’. In contrast with employers thinking people with disabilities are not productive, some employers stated that people because of their disabilities have unique advantages in organizations (2%). To illustrate, some employers mentioned that people with disabilities could offer products without mistakes [46]. We also found that factors can differ depending on the type of disability. Some employers for instance mentioned that people in a wheelchair were turned down for jobs because the nature of the job required prolonged standing and it would be very costly to adapt the working environment [43]. Nevertheless, we found that most studies researched disability in general (59%). This means that these studies did not focus on one specific type of disability. Other studies focused on one type of disability (37%) or specified multiple types of disability (6%). For each factor we coded if variability between disabilities was reported or if studies researched disability in general. We found that variability was often unknown (65% of cases). In other words, most studies did not differentiate between disabilities.

## Discussion

This review aimed to map *which capabilities, opportunities and motivations of employers impact whether they hire people with disabilities.* We have systematically investigated capabilities, opportunities and motivations (COM) that influence hiring behaviors (B) as reported in scientific studies. We found (1) most studies measure hiring behavior subjectively; (2) 32 factors based on the COM-B model that affect hiring behavior; (3) factors can have mixed effects on hiring behavior, meaning they can have a positive, negative or no effect. Our findings contribute both theoretically and methodologically to the literature and result in a research agenda for future research on employers’ hiring behavior.

First, hiring behavior of employers is rarely measured objectively. It is rather measured by subjective reports of employers that vary from reflecting about factors that influence employers across society to estimating hypothetical hiring of people with disabilities. This can be problematic because subjective reports of hiring do not always represent actual hiring, which harms our understanding of employers’ hiring behavior. Future research should attempt to use organizational data to objectively measure hiring behavior. This finding is in line with a bigger trend in research where studies using objectively measured behaviors are becoming more important [54]. On top of that, experimental methodology is rarely used. Experimental methodology, however, can be beneficial to detect causal relationships [55]. This is a valuable avenue for future research because it would allow researchers to dissect what causes employers to (not) hire people with disabilities rather than state correlations. Ideally, we would use field experiments to research what influences hiring behavior in real life settings. However, experimental methods such as survey experiments can also measure proxies of behavior by for instance letting an employer hypothetically hire a person.

Second, we found 32 different factors that can influence employers’ hiring behavior of people with disabilities. These factors are spread across most dimensions of the COM-B model. This finding supports our idea that hiring behavior is the result of a complex decision-making process that includes capabilities, opportunities as well as motivations. Moreover, it shows how the COM-B model can be a useful framework for gaining insight into how to stimulate employers to hire more employees with a disability. The most mentioned barriers were employers’ beliefs about people with disabilities not being productive (14%) and their expectations about costs (11%), and lacking knowledge about disabilities (5%). The most mentioned facilitators were pro-social motivation (5%), working in a large organization (4%) and expecting a competitive advantage (4%). Believing people with disabilities are not productive is related to attitudes about people with disabilities which is often discussed in literature [14]. Working in a large organization and expectations about costs are also mentioned in overview articles [18]. The other factors, such as lacking knowledge about disabilities, expecting a competitive advantage and pro-social motivation have—to date—been largely overlooked in overview studies. Future research is especially needed into the conditions under which these factors influence hiring behaviors. It has to be noted that expecting a competitive advantage might occur in this review because over the last decade, attention for on positive aspects of hiring people with disabilities is growing [19].

Future research will need to use validated concepts and scales. This can be beneficial because, first, it supports scholars’ communication with one another with can lead to theory building. Second, it allows us to draw on other streams of literature that explains the origin, causes and intervention possibilities related to these variables. Organizational psychology and economics for instance have a long tradition of literature on pro-social motivation while public administration research has elaborated knowledge on administrative burden [32, 44, 45].

An important benefit of using the COM-B framework is that the identification of capabilities, opportunities and motivations also indicates which behavioral interventions might be beneficial for influencing hiring behaviors through using the Behavior Change Wheel [9]. For example, for capabilities, information about different disability types and what to expect can be helpful. Here, innovations such as immersing virtual reality experiences could help people to understand conditions such as autism [56]. For opportunities, targeting especially small companies with additional resources can increase hiring of people with disabilities. For motivation, sharing positive experiences in working with people with disabilities is a storytelling intervention aimed to influence employers’ attitudes. Employers for instance mentioned that having seen a person with a disability perform well has led them to believe that people with disabilities could be productive [57].

Third, we showed that some factors can have no effect, as well as a positive or negative effect on hiring people with disabilities. For instance, ‘believing co-workers will respond negatively’ can negatively influence hiring behavior (82%), but can also have no effect on hiring whatsoever (18%). This suggests that there may be contextual explanations why a factor sometimes does and sometimes *does* *not* impact hiring of people with disabilities. We have identified three possible explanations worth further investigation. The first explanation is company size. Studies in our sample indicate that while small companies might be very concerned about costs, while this is less important for large companies [49, 58]. Small companies can also have more problems with administrative burdens or limited internal resources [46]. Future research could explicitly compare the hiring behavior of employers of large and small companies.

The second explanation is pro-social motivation. We find that pro-social motivation might be a reason to ‘push through’ certain barriers for hiring people with disabilities. For instance, studies show that the lack of available positions can have negative effects on hiring [59]. Sometimes this lack of available positions has, however, no effect as positions were tailored to individuals [60]. This happened because employers were motivated to include people with disabilities in their organization due to their mission. Then, when positions did not match, they decided to find a better match, and not give up. This indicates that for some employers, hiring people with disabilities is important regardless of barriers such as available positions. Future research could explicitly study pro-social motivation of employers and test interventions to amplify pro-social motivation if present.

Third, institutional and, specifically, policy contexts matter as well in explaining the different impact that factors may have on hiring behavior of employers. For example, cost concerns may be alleviated by specific policy programs such as wage subsidies or subsidies for work accommodations. Another example is that labor shortages might stimulate employers to hire more people with disabilities. Related to this, we found that most studies do not differentiate and/or compare different types of disability when studying hiring behavior. This is surprising because there is a great range of disabilities people can have. We, indeed, find that the effect of factors for hiring behavior *can* differ per disability type. For instance, Kocman et al. [59] report that employers believed that customers would react more negatively to people with intellectual disabilities than people with physical disabilities. Moreover, Notaroberto and d’Angelo [61] reported that employers have a preference for disabilities which require less changes in the physical environment. Future research would benefit from comparing hiring behavior of employers for people with different types of disabilities, as well as hiring under different contextual circumstances, such as when there is a shortage of personnel.

## Limitations

This study, like any, inevitably has limitations. First, we used a novel method based on machine learning, ASReview, to select studies from databases. The method helps to identify eligible studies in an efficient and transparent way. However, we can never be sure that we have not missed any studies. On top of that, our review might have missed articles due to our choice of databases. This would, however, be the case with any systematic review. We provide open data on OSF to ensure full transparency of this process (see https://osf.io/av95r/).

This systematic review gives insight into effects by coding for no, negative or positive effects on hiring behavior as reported in literature. Because of the heterogeneity of factors and different methodologies included, a meta-analysis was not the aim of this study. We did however include a measure to indicate the direction of the effect reported. This measure of effect is limited as it does not include statistical measures, in the case of qualitative studies, and effect sizes. Future studies can focus on one study type and one factor to explore their effects.

We used the COM-B model, and underlying, the TDF to code factors into categories. We found that using this model functioned well to identify which factors to include and how to understand them. Nevertheless, sometimes it was unclear whether factors belonged to one clear-cut category. We solved this by having two coders discuss their applicability for each category. For future research, we recommend having at least two coders decide about the application of factors were possible and pre-registering the review if possible.

## Conclusion

We conclude that many different capabilities, opportunities and motivations exist that influence hiring behavior of employers regarding people with disabilities. Many of these factors offer opportunities for interventions that can stimulate employers to hire more people with disabilities. The top three *barriers* to hiring people with disabilities as reported in literature are (1) believing people with disabilities are not productive, (2) expecting costs and (3) lacking knowledge about disabilities. The top three *facilitators* to hire people with disabilities are (1) having pro-social motivation, (2) working in a large organization and (3) expecting competitive advantages. Other factors such as lacking intentions to hire and feeling in control were found to be rarely researched. Identifying their effects on hiring behaviors therefore requires future research. All factors were found to have the potential to have different effects on employers’ hiring behavior under different circumstances. Changing these behaviors will therefore require a multifaceted approach that has to possibly vary per organization or group of employers.

## Supplementary Information

Below is the link to the electronic supplementary material.

Supplementary file1 (DOCX 15 kb)

Supplementary file2 (DOCX 15 kb)

Supplementary file3 (DOCX 17 kb)

Supplementary file4 (DOCX 22 kb)

## Data Availability

The datasets generated during and/or analysed during the current study are available in the OSF repository under https://osf.io/av95r/.
