# Supplementary material for: Why Do Employers (Fail to) Hire People with Disabilities? A Systematic Review of Capabilities, Opportunities and Motivations
Source: J Occup Rehabil. 2023 Jan 23;33(2):329–40. doi: 10.1007/s10926-022-10076-1 (PMC10172218; doi:10.1007/s10926-022-10076-1)
Supplement: Supplementary file 1 — Supplementary file1 (DOCX 15 kb) [file 10926_2022_10076_MOESM1_ESM.docx]

Supplement A

| **Table 5** *The Theoretical Domains Framework* *as derived from Cane et al. [30]* | |
| --- | --- |
| Domain | Definition [possibly relevant constructs] |
| 1. Knowledge | An awareness of the existence of something [procedural knowledge, knowledge of task environment]. |
| 2. Skills | An ability or proficiency, acquired through practice [skills, skills development, competence, ability, interpersonal skills, practice, skill assessment] |
| 3. Social/Professional Role and Identity | A coherent set of behaviors and displayed personal qualities of an individual in a social or work setting [professional identity, professional roles, social identity, identity, group identity, leadership, professional boundaries, professional confidence, organizational commitment] |
| 4. Beliefs about Capabilities | Acceptance of the truth, reality, or validity about an ability, talent, or facility that a person can put to constructive use [perceived competence, self-esteem, self-efficacy, self-confidence, empowerment, perceived behavioural control, professional confidence]. |
| 5. Optimism | The confidence that things will happen for the best or that desired goals will be attained [optimism, pessimism, unrealistic optimism, identity]. |
| 6. Beliefs about Consequences | Acceptance of the truth, reality, or validity about outcomes of a behavior in a given situation [outcome expectancies, anticipated regret, consequences]. |
| 7. Reinforcement | Increasing the probability of a response by arranging a dependent relationship, or contingency, between the response and a given stimulus [incentives, punishment, consequences, reinforcement]. |
| 8. Intentions | A conscious decision to perform a behavior or a resolve to act in a certain way [implementation intention, stages of change model] |
| 9. Goals | Mental representations of outcomes or end states that an individual wants to achieve [goal setting, action planning, goal priorities] |
| 10. Memory, Attention and Decision Processes | The ability to retain information, focus selectively on aspects of the environment and choose between two or more alternatives [tiredness, memory, attention, cognitive overload, attention, decision-making] |
| 11. Environmental Context and Resources | Any circumstance of a person's situation or environment that discourages or encourages the development of skills and abilities, independence, social competence, and adaptive behavior [resources, environmental stressors, organizational culture, salient events, barriers and facilitators, person x environment interaction] |
| 12. Social influences | Those interpersonal processes that can cause individuals to change their thoughts, feelings, or behaviors [social norms, group conformity, social comparisons, group norms, social support, power] |
| 13. Emotion | A complex reaction pattern, involving experiential, behavioural, and physiological elements, by which the individual attempts to deal with a personally significant matter or event [fear, anxiety, affect, stress, depression, burn-out]. |
| 14. Behavioural Regulation | Anything aimed at managing or changing objectively observed or measured actions [self-monitoring, breaking habit, action planning] |
