# Supplementary material for: Why Do Employers (Fail to) Hire People with Disabilities? A Systematic Review of Capabilities, Opportunities and Motivations
Source: J Occup Rehabil. 2023 Jan 23;33(2):329–40. doi: 10.1007/s10926-022-10076-1 (PMC10172218; doi:10.1007/s10926-022-10076-1)
Supplement: Supplementary file 2 — Supplementary file2 (DOCX 15 kb) [file 10926_2022_10076_MOESM2_ESM.docx]

Supplement B

| **Table 6**  *Searches in databases* | | | |
| --- | --- | --- | --- |
| Date | Search engine | Search terms | Limit |
| 7-4-2021 | Web of Science | (TI=((Hir* or employ* or selection or recruit* or job*) and (Disabl* or disabil* or handicap* or impair* or disorder*) )) AND LANGUAGE: (English) | timespan 2010-2021 |
| 6-4-2021 | PsychInfo | ((Hir* or employ* or selection or recruit* or job*) and (Disabl* or disabil* or handicap* or impair* or disorder*)).ti. | limit to (english language and yr="2010 -Current") |
| 7-4-2021 | Scopus | ( TITLE ( hir* OR employ* OR selection OR recruit* OR job* ) AND TITLE ( disabl* OR disabil* OR handicap* OR impair* OR disorder* ) ) | timespan 2010-2021 |
| 6-4-2021 | PubMed | ( (disabl*[Title] OR disabil*[Title] OR handicap*[Title] OR impair*[Title] OR disorder*[Title])) AND (hiring[Title] OR hire[Title] OR employ*[Title] OR selection[Title] OR recruit*[Title] OR job[Title]) | timespan 2010-2021 |

| **Table 7**  *Searches in journals* | | | |
| --- | --- | --- | --- |
| **Date** | **Journal** | **Search terms** | **Limit** |
| 4-1-2022 | Journal of Vocational Rehabilitation | ((Hir* OR employ* OR selection OR recruit* OR job*) AND (Disabl* OR disabil* OR handicap* OR impair* OR disorder*)) | 2010 to 2022 |
| 4-1-2022 | Journal of Occupational Rehabilitation | (Hir* OR employ* OR selection OR recruit* OR job*) AND (Disabl* OR disabil* OR handicap* OR impair* OR disorder*) | 2010 to 2022 |
| 4-1-2022 | Rehabilitation Counseling Bulletin | for [[All hir*] OR [All employ*] OR [All selection] OR [All recruit*] OR [All job*]] AND [[All disabl*] OR [All disabil*] OR [All handicap*] OR [All impair*] OR [All disorder*]] | 2010 to 2022 |
