# Supplementary material for: Why Do Employers (Fail to) Hire People with Disabilities? A Systematic Review of Capabilities, Opportunities and Motivations
Source: J Occup Rehabil. 2023 Jan 23;33(2):329–40. doi: 10.1007/s10926-022-10076-1 (PMC10172218; doi:10.1007/s10926-022-10076-1)
Supplement: Supplementary file 3 — Supplementary file3 (DOCX 17 kb) [file 10926_2022_10076_MOESM3_ESM.docx]

Supplement C

| **Table 7**  *Definitions of the variables found in the systematic review* | | |
| --- | --- | --- |
| Variable | Definition | % recoded |
| Being willing to take a risk | Employers are willing to take a risk when hiring people with disabilities. | 50% |
| Believing co-workers will respond negatively | Employers believe that co-workers will respond negatively to hiring people with disabilities. | 9% |
| Believing customers will respond negatively | Employers believe that customers will respond negatively to hiring people with disabilities. | 0% |
| Believing people with disabilities are not productive | Employers believe that people with disabilities are not equally productive to others. | 16% |
| Believing people with disabilities have unique advantages | Employers believe that people with disabilities have unique qualities that are beneficial for the organization, such as the ability to deliver zero-error products. | 0% |
| Believing stereotypes | Employers have negative stereotypes about people with disabilities | 18% |
| Complying with laws | Employers are complying with laws concerning the hiring of people with disabilities. | 8% |
| Expecting a competitive advantage | Employers expect a competitive advantage as a result of hiring people with disabilities, for instance by strengthening their reputation. | 6% |
| Expecting costs | Employers are expecting imagined or real costs as a result of hiring people with disabilities. | 0% |
| Expecting financial gains | Employers expect financial gains as a consequence of hiring people with disabilities, for instance through | 0% |
| Expecting limited consequences of hiring | Employers expect almost no consequences when they hire a person with a disability. | 0% |
| Expecting negative safety consequences | Employers believe that people with disabilities will create unsafe situations on the work floor | 0% |
| Experiencing administrative burden | Employers experience administrative burden when hiring people with disabilities. Administrative burdens are burdens that employers experience when interacting with the state (Moynihan, Herd, and Harvey 2015). | 26% |
| Experiencing negative emotions | Employers experience negative emotions when encountering people with disabilities. | 0% |
| Experiencing positive emotions | Employers experience positive emotions when encountering people with disabilities. | 0% |
| Feeling in control | Employers feel in control when hiring people with disabilities. | 0% |
| Getting financial incentives | Employers are receiving financial support when hiring people with disabilities, for instance through subsidies. | 17% |
| Having positions available | Employers have job positions available for people with disabilities. | 50% |
| Having pro-social motivation | Employers have pro-social motivation. Pro-social motivation is defined as the desire to contribute to the well-being of others (Grant and Berg 2021). | 0% |
| Having support from within the organization | Employers experience support from within the organization, for instance through commitment from managers. | 60% |
| Lacking external support | Employers experience limited support from outside their organization, for instance by governments or vocational rehabilitation services. | 47% |
| Lacking intentions to hire | Employers do not have the intention to hire people with disabilities. | 33% |
| Lacking knowledge about disabilities | Employers lack knowledge about what disabilities are. | 43% |
| Lacking knowledge about recruiting | Employers do not know how to recruit people with disabilities. | 14% |
| Not encountering qualified people with disabilities applying | Employers do not see people with disabilities applying for jobs within their organizations. | 0% |
| Not knowing how to manage people with disabilities | Employers do not know how to manage people with disabilities. | 40% |
| Working for a public organization | Employers work for an organization that is public. Public organizations are government organizations funded by public money or with societal goals. This category also includes federal contractors. | 0% |
| Working in a large organization | Employers are working within a large organization where many other people work. | 0% |
| Working in a worksite with physical barriers | Employers are working within a worksite with physical barriers, such as no elevator or wheelchair ramp. | 14% |
| Working in an organization with a policy for inclusion | Employers are working in organizations that have policy for inclusion, such as | 8% |
| Worrying about legal consequences | Employers are worried that hiring people with disabilities will bring on difficult legal scenarios. | 0% |
| Worrying about spending more time to assist | Employers are worried that people with disabilities will need much more time for support. | 0% |
